# Targeted next generation sequencing of endoscopic ultrasound acquired cytology from ampullary and pancreatic adenocarcinoma has the potential to aid patient stratification for optimal therapy selection

**DOI:** 10.18632/oncotarget.9440

**Published:** 2016-05-18

**Authors:** Ferga C. Gleeson, Sarah E. Kerr, Benjamin R. Kipp, Jesse S. Voss, Douglas M. Minot, Zheng Jin Tu, Michael R. Henry, Rondell P. Graham, George Vasmatzis, John C. Cheville, Konstantinos N. Lazaridis, Michael J. Levy

**Affiliations:** ^1^ Division of Gastroenterology & Hepatology, Mayo Clinic Rochester, MN, USA; ^2^ Department of Laboratory Medicine & Pathology, Mayo Clinic Rochester, MN, USA; ^3^ Division of Biomedical Statics & Informatics, Department of Health Sciences Research, Mayo Clinic Rochester, MN, USA; ^4^ Center for Individualized Medicine, Mayo Clinic, Rochester, MN, USA

**Keywords:** endoscopic ultrasound fine needle aspiration, pancreatic adenocarcinoma, targeted next-generation sequencing, mutation concordance, personalized medicine

## Abstract

**Background & Aims:**

Less than 10% of registered drug intervention trials for pancreatic ductal adenocarcinoma (PDAC) include a biomarker stratification strategy. The ability to identify distinct mutation subsets via endoscopic ultrasound fine needle aspiration (EUS FNA) molecular cytology could greatly aid clinical trial patient stratification and offer predictive markers. We identified chemotherapy treatment naïve ampullary adenocarcinoma and PDAC patients who underwent EUS FNA to assess multigene mutational frequency and diversity with a surgical resection concordance assessment, where available.

**Methods:**

Following strict cytology smear screening criteria, targeted next generation sequencing (NGS) using a 160 cancer gene panel was performed.

**Results:**

Complete sequencing was achieved in 29 patients, whereby 83 pathogenic alterations were identified in 21 genes. Cytology genotyping revealed that the majority of mutations were identified in KRAS (93%), TP53 (72%), SMAD4 (31%), and GNAS (10%). There was 100% concordance for the following pathogenic alterations: KRAS, TP53, SMAD4, KMT2D, NOTCH2, MSH2, RB1, SMARCA4, PPP2R1A, PIK3R1, SCL7A8, ATM, and FANCD2. Absolute multigene mutational concordance was 83%. Incremental cytology smear mutations in GRIN2A, GATA3 and KDM6A were identified despite re-examination of raw sequence reads in the corresponding resection specimens.

**Conclusions:**

EUS FNA cytology genotyping using a 160 cancer gene NGS panel revealed a broad spectrum of pathogenic alterations. The fidelity of cytology genotyping to that of paired surgical resection specimens suggests that EUS FNA represents a suitable surrogate and may complement the conventional stratification criteria in decision making for therapies and may guide future biomarker driven therapeutic development.

## INTRODUCTION

Conventional non-targeted chemotherapy regimens are the standard of care for patients with ampullary (AA) and pancreatic adenocarcinoma (PDAC). Yet, clinical trials frequently offer targeted agents to patients without screening for actionable pathogenic alterations. Less than 10% of registered drug intervention trials for PDAC include a biomarker stratification strategy. [1] The five most commonly mutated genes of PDAC in the Catalogue of Somatic Mutations in Cancer (COSMIC) database are KRAS (71%), TP53 (49%), CDKN2A (22%), SMAD4 (20%), and ARID1A (6%). [1] In 2014, the National Cancer Institute (NCI) created a scientific framework for PDAC with 4 initiatives. [2] The objective of one of these initiatives was to expand PDAC research to develop new approaches that interfere with RAS oncogene dependent signaling pathways. RAS can activate several downstream effectors, including the PI3K-AKT-mTOR and the RAS-RAF-MEK-ERK pathways, which are involved in cell survival and proliferation.

Evolving novel therapeutic strategies include agents that not only target the tumor itself, but also the tumor microenvironment. Such unique or combined approaches include vaccine based immunotherapy, the use of stromal depleting agents, BRCA related therapies, inhibitors of autophagy, angiogenesis inhibition and NOTCH signaling pathway inhibitors. [3–5] Genetic signatures could be used to direct personalized PDAC treatment in the future. [6]

The ability to identify distinct mutation subsets via endoscopic ultrasound fine needle aspiration (EUS FNA) molecular cytology could greatly aid clinical trial patient stratification for optimal therapy selection and offer predictive markers. Such a development would represent a crucial step in the field of personalized medicine. It has been observed that tumors characterized by a concurrent *TP53* and *SMAD4* wild type status have an indolent behavior and an improved therapy response with low metastatic potential. [7, 8] However, *TP53* mutant tumors accompanied by a *SMAD4* wild type profile exhibit increased metastatic potential, and finally a combination of *TP53* and *SMAD4* mutations represent the most aggressive and widespread metastatic PDAC. Therefore, stratification of cytotoxic agent eligible patients using EUS FNA specimens to determine the multigene mutation status may be of prognostic benefit and facilitate appropriate tumor specific targeted therapy.

The aims of our translational study using a commercially available 160 gene targeted NGS comprehensive cancer panel were to determine among a cohort of patients with chemotherapy treatment naïve AA and PDAC the 1.) multigene mutational landscape within EUS FNA cytology smear specimens, 2.) spectrum of functional gene groupings, 3.) frequency and subtype of *KRAS, TP53* and *SMAD4* pathogenic alterations and 4.) multigene mutation concordance between paired EUS FNA cytology smears and surgical resection specimens.

## RESULTS

### Clinical demographics

Targeted NGS was achieved in 29 chemotherapy naïve patients [65.4 ± 13.4 years, male gender n=19 (65.5%)]. Three (10.3%) patients were < 45 years of age at the time of diagnosis. A family history (≥ 1 first degree relative) of PDAC was notable for 3 (10.3%) patients. Smoking status at the time of FNA included never smokers [n=13 (44.8%)] and ex or current smokers [n=16 (55.3%)] with a 13.5 (5-20) pack year history. The Ca19-9 level at diagnosis was 88 (30-260) U/mL [normal < 55U/mL: n=17 (58.6%) > 55 U/mL]. The fasting serum glucose level was 112 (96-153.5) mg/dL (normal 70-100 mg/dL), 17 (62.1%) of whom had a level > 100 mg/dL at diagnosis.

### Tumor demographics

The cohort was comprised of the following: PDAC [n=21 (72.4%); 20 primary and 1 recurrent], AA [n=4 (13.8%)], malignant transformation of intraductal papillary mucinous neoplasm (IPMN) [n=3 (10.3%)], and Lynch Syndrome associated PDAC [n=1 (3.4%)]. The tumors were located within the pancreatic: head [n=19 (65.5%)], body [n=5 (17.2%)], ampulla [n=4 (13.8%)], and complete gland infiltration [n=1 (3.4%)]. The median tumor size at EUS was 3cm (2.5-4.1). The spectrum of subsequent surgical intervention included the following: pancreaticoduodenectomy [n=20 (69%)], distal pancreatectomy [n=5 (17.2%)], total pancreatectomy [n=3 (10.3%)], and a completion pancreatectomy for a patient with recurrent disease [n=1 (3.4%)].

### Disease recurrence and mortality

Disease recurrence developed in 17 (58.6%) patients at 13 (7.7-21.5) months following EUS FNA. The initial site of tumor recurrence was liver [n=6 (35.3%)], peritoneum [n=5 (29.4%)], lung [n=2 (11.8%)], malignant ascites [n=2 (11.8%)], a new primary [n=1 (5.9%)], and a cutaneous chest drain site [n=1 (5.9%)]. Twenty (68.9%) patients died at a median of 17.8 (10.9-32.6) months. Overall follow up from the time of EUS FNA to mortality or to the end of the study was 22.9 (10.9-42.6) months.

### Cytology genotyping using a commercially available comprehensive cancer panel

Targeted NGS sequencing revealed that 83 pathogenic alterations were identified in 21 genes. (Supplementary Table S1) Patients harbored a median of 2 (2-3.5) pathogenic alterations per tumor. Twenty-seven (93.1%), 13 (44.8%) and 6 (20.7%) patients harbored ≥ 2, ≥ 3 and ≥ 4 pathogenic alterations per tumor, respectively. Genotyping revealed that the majority of mutations were identified in *KRAS* (93.1%), *TP53* (72.4%), *SMAD4* (31%), and *GNAS* (10.3%). (Table 1) All patients with *GNAS* mutations had concurrent *KRAS* and *TP53* co-mutations. No somatic P16 mutations were identified. Based on currently available chemotherapeutic agents, no “actionable” or “druggable” mutations were identified in *BRAF, PIK3CA, BRCA, PALB2, ERBB1, MET, FGFR1*, or *EGFR.*

**Table 1:** EUS FNA pathogenic alteration spectrum in 29 patients

	Gene	Number of patients	Mutation frequency
**1**	KRAS	27	93.1%
**2**	TP53	21	72.4%
**3**	SMAD4	9*	31%
**4**	GNAS	3	10.3%
**5**	ARID1a	2**	6.9%
**6**	NOTCH2	2	6.9%
**7**	KMT2D	2	6.9%
**8**	KDM6A	2	6.9%
**9**	HNF1A	1	3.5%
**10**	CARD11	1	3.5%
**11**	SMARCA4	1	3.5%
**12**	PPP2R1A	1	3.5%
**13**	PIK3R1	1	3.5%
**14**	SCL7A8	1	3.5%
**15**	MSH2	1	3.5%
**16**	RB1	1	3.5%
**17**	ATM	1	3.5%
**18**	FANCD2	1	3.5%
**19**	FBXW7	1	3.5%
**20**	GATA3	1	3.5%
**21**	GRINDA	1	3.5%

Of 29 patients:

* 1 patient had 2 SMAD4 alterations:

** 1 patient had 2 ARID1A alterations

### Characterization of the KRAS mutational profile

**Figure 1:**
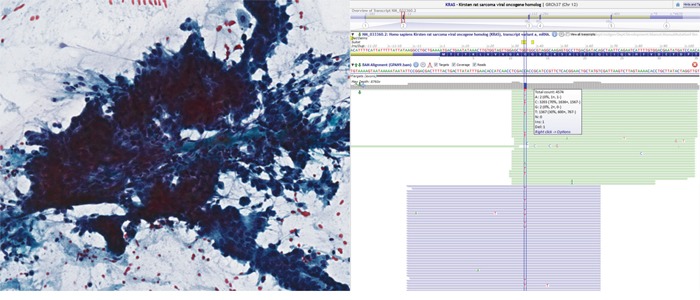
Pap-stained cytology slide (left) from pancreatic adenocarcinoma The sheet of cells shows loss of polarity, crowding and overlapping nuclei. Representative sequencing results (right) in Alamut showing KRAS c.35G>A, p. Gly12Asp missense mutation in 30% of alleles. Note that the reverse strand was sequenced suggesting a C>T mutation in the figure.

The observed *KRAS* mutations were composed of alterations in codons 12, 13 and 61 in 85.2%, 3.7% and 11.1%, respectively. The most frequently identified genotypes were Gly12Val (33%) and Gly12Asp (33%) (Figure 1). Alterations in codons 13 and 61 were only identified in either current or former smokers. Overall survival for patients with a *KRAS* mutation was 24.2 (13.8-44.6) months. Neither Gly12Val (HR = 0.6292; 95% CI 0.2505 to 1.5807; p=0.36) or Gly12Asp (HR = 0.9956; 95% CI 0.3829 to 2.5887; p=0.99) status was associated with disease related mortality. Two *KRAS* wild-type (WT) patients harbored mutations in *TP53* and *FBXW7*, respectively.

### Characterization of the TP53 mutational profile

Twenty-one patients harbored 20 *TP53* mutations including 15 missense mutations, 2 nonsense mutations, 1 splice site mutation, 1 in-frame deletion and 1 single base pair duplication resulting in a premature stop codon (Supplementary Table S2). TP53 was the second most frequently identified pathogenic alteration in patients, 19 (90.5%) and 5 (23.8%) of whom had either a *KRAS* or *SMAD4* co mutation.

### Characterization of the SMAD 4 mutational profile

Ten *SMAD 4* pathogenic alterations, the third most common alteration in the cohort, were observed in 9 (31%) patients, but in no patient with AA. SMAD 4 genotyping revealed a heterogeneous group of pathogenic alterations to include: p. Arg361Cys, p. Trp524Cys, p. Gln256Ter, p. Ala406Val x 2, p. His530ThrfsTer47, p. Arg135Ter, p. Asp351del, p. Asp351GlyfsTer27, and p. Tyr114IlefsTer7, respectively. There were no clinical demographic differences between SMAD 4 mutant and WT patients to include: age, gender, first degree relative with PDAC, smoking status, fasting glucose status, baseline Ca19-9 level, tumor location, size or stage, disease recurrence or mortality (Table 2). All patients had concurrent *KRAS* mutations.

**Table 2:** SMAD4 mutant population clinical demographics and pathogenic alteration status

	SMAD 4 mutant N=9	SMAD 4 WT N=20	P value
Age (years)	63.1 ± 14.2	66.2± 13.3	0.574
Gender (male)	6 (66.7%)	13 (65%)	0.9
Positive Family History (1^st^ degree relative)	1 (11.1%)	2 (10%)	0.9
Current Smoker	6 (66.7%)	10 (50%)	0.4543
Ca 19-9 (U/mL)	689 ± 1,398.7	146.7 ± 159.6	0.0915
Fasting glucose (mg/dL)	139.3 ± 70.1	124.2± 36.5	0.4486
Elevated glucose (> 100 mg/dL)	8 (88.9%)	9 (45%)	0.0432
Location (head)	5 (55.6%)	14 (70%)	0.6749
Size (cm)	4.5 ± 3.2	3.3 ± 1.7	0.1953
TNM ≥ T3N0	9 (100%)	13 (65%)	0.0661
Stage Grouping ≥ 2b	6 (66.7%)	14 (70%)	0.9
Perineural Invasion	6 (66.7%)	7 (35%)	0.2256
R0 Resection Status	9 (100%)	17 (85%)	0.5320
Disease Recurrence	7 (77.8%)	10 (50%)	0.2341
Mortality	7 (77.8%)	13 (65%)	0.6749
Time to Mortality (months)	18.3 ± 8.5	20.3 ±14.7	0.7082
Progression Free Survival	2 (2%)	6 (30%)	0.9
KRAS mutant status	9(100%)	17 (85%)	0.5320
P53 mutant status	5 (55.6%)	17 (85%)	0.1581
KDM6A mutant status	2 (22.2%)	0 (0%)	0.0887
≥ 2 pathogenic alterations/tumor	9 (100%)	17 (85%)	0.5320
≥ 3 pathogenic alterations/tumor	8 (88.9%)	4 (20%)	0.0009
≥ 4 pathogenic alterations/tumor	3 (33.3%)	3 (15%)	0.3391

### Multigene mutational concordance with paired surgical pathology specimens

In parallel with the cytology smear specimens, matched site surgical pathology specimens of 18 patients, who were selected as they had sufficient material for a multigene mutation concordance evaluation [64.6 ± 12.0 years; male gender n=11 (61.1%); Ca19-9 level: 59 (30-186) U/mL; fasting glucose (mg/dL): 112.5 (96-143)] also underwent targeted NGS with the Qiagen™ Human Comprehensive Cancer GeneRead DNAseq Targeted Array V2.

**Figure 2:**
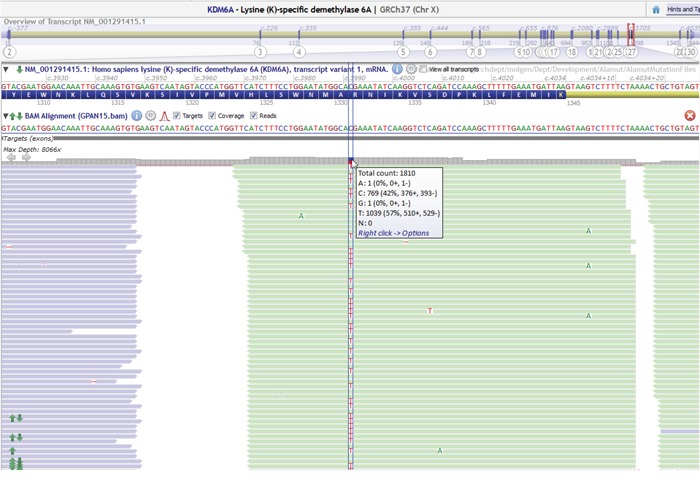
Example of a sequencing data in Alamut displaying nonsense mutation (c.3991C>T, p. Arg1331X) in KDM6A with an allele frequency of 57%

Paired cytology NGS with matched surgical pathology NGS patients revealed 56 and 50 pathogenic alterations, respectively, in 19 and 16 genes. Five (27.8%) patients had ≥ 4 pathogenic alterations identified per tumor. *KRAS* (94.4%), *TP53* (66.7%) and *SMAD 4* (38.9%) were the most frequently identified pathogenic alterations. Fifteen of 18 (83.3%) paired patients had absolute multigene mutational concordance (Table 3). There was 100% concordance for the following individual pathogenic alterations: *KRAS, TP53, SMAD4, KMT2D, NOTCH2, MSH2, RB1, SMARCA4, PPP2R1A, PIK3R1, SCL7A8, ATM*, and *FANCD2*. Mutations in *GRIN2A* (p. Val1197Met) (allele frequency = 14%), *GATA3* (p. Ala102Thr) (allele frequency = 6%), *GNAS* (p.Gly282Ser) (allele frequency = 5%) and *KDM6A* (p. Gln524Ter) (allele frequency = 7%) were only identified within the EUS FNA cytology specimens from three patients despite careful re-examination of the of the raw sequence reads in the resection specimens (Figure 2). The estimated tumor % in these 2 patients for FFPE versus cytology specimens were similar (70% vs. 70% and 70% vs. 30%, respectively). Overall, the average mutant allele frequencies in paired FFPE versus cytology samples with concordant mutations were 35% versus 34%, also suggesting similar tumor percentage between FFPE and cytology samples.

**Table 3:** Paired concordance assessment of EUS FNA cytology to matched surgical pathology

Patient	primary pathology	stage grouping	Cytology	surgical pathology	Concordance
**1**	PDAC	Ib	KRAS, SMAD4, ATM	KRAS, SMAD4, ATM	100%
**2**	PDAC	IIa	KRAS, SMAD4	KRAS, SMAD4	100%
**3**	PDAC	IIb	TP53, SMAD4	TP53, SMAD4	100%
**4**	PDAC	IIb	KRAS, SMAD4	KRAS, SMAD4	100%
**5**	IPMN	IIa	TP53, KRAS, SMAD4	TP53, KRAS, SMAD4	100%
**6**	PDAC	IIb	TP53, KRAS	TP53, KRAS	100%
**7**	PDAC	Ia	TP53, KRAS	TP53, KRAS	100%
**8**	PDAC	IIb	TP53, KRAS	TP53, KRAS	100%
**9**	PDAC	IIb	TP53, KRAS, SMARCA4, PPP2R1A	TP53, KRAS, SMARCA4, PPP2R1A	100%
**10**	PDAC	IIb	TP53, KRAS, SCL7A8	TP53, KRAS, SCL7A8	100%
**11**	PDAC	IIb	TP53, KRAS	TP53, KRAS	100%
**12**	PDAC	IIb	TP53, KRAS, GNAS, CARD11, RB1	TP53, KRAS, CARD11, RB1	80%
**13**	Lynch associated PDAC	Ia	TP53, KRAS, GNAS, NOTCH2, MSH2, ARID1a	TP53, KRAS, GNAS, NOTCH2, MSH2, ARID1a	100%
**14**	PDAC	IV	TP53, KRAS, SMAD4, KDM6A, ARID1a (x 2), PIK3R1, GATA3	TP53, KRAS, SMAD4, PIK3R1	57%
**15**	PDAC	IIa	KRAS, SMAD4, KMT2D, FANCD2	KRAS, SMAD4, KMT2D, FANCD2	100%
**16**	PDAC	IIb	KRAS, NOTCH2	KRAS, NOTCH2	100%
**17**	AA	IIa	KRAS, GRINDA	KRAS	50%
**18**	PDAC	III	TP53, KRAS, SMAD4	TP53, KRAS, SMAD4	100%

## DISCUSSION

Pancreatic ductal and ampullary adenocarcinomas are biologically heterogeneous tumors. Detailed global genomic analyses have identified that there are 12 core signaling pathways and 16 genes significantly genetically altered in the majority of PDAC patients. [10–11] We have previously reported a 50 gene mutation assessment of a spectrum of other primary, locally advanced and metastatic diseases using EUS FNA cytology specimens. [9, 12–16] Our newly presented data represent a multigene mutation assessment of 160 cancer associated genes using targeted NGS. This unique assessment further exemplifies the collective genetic diversity of PDAC - AA and may be used to identify specific molecular patient cohorts most likely to benefit from agents designed to target specific pathways or genomic features. [17]

The ability to apply targeted NGS to routine EUS FNA cytology offers tremendous promise for such an endeavor in an evolving era of individualized medicine. We have observed a broad spectrum of pathogenic alterations in 21 genes, whereby 93% of patients had ≥ 2 pathogenic alterations per tumor. This further highlights the clear need for customized combination therapy with the goal of enhancing therapeutic response and patient outcome.

The performance of KRAS mutation analysis in EUS FNA pancreas mass specimens from a variety of molecular techniques is well documented. [18–23] However, we report that an EUS FNA molecular cytology assessment with targeted NGS has the ability to identify and dissect KRAS mutation subtypes as part of a multigene mutation analysis. Furthermore, EUS FNA identified that 31% of our cohort harbored a SMAD4 mutation and they were more likely to harbor additional pathogenic alterations when compared to their WT counterparts. We did not observe currently “druggable” mutations within the evaluated cohort as the following were of wild type status: BRAF, PIK3CA, BRCA, PALB2, ERBB1, MET, FGFR1, and EGFR. Nevertheless, our discoveries could be applied to prospective clinical trials and aid a combined phenotypic-genotypic approach to facilitate the development of precision medicine.

These are the first data from a commercial 160 cancer gene panel demonstrating that EUS FNA cytology specimens provide an ideal surrogate to surgical specimens for detecting pathogenic alterations associated with PDAC and AA. This information is key given the narrow cohort that would undergo a surgical biopsy and the necessity for pre-operative delivery of personalized tumor specific care. Interestingly, we identified that the observed cytology mutational spectrum was broader than that of the corresponding surgical pathology specimens. Mutations in GRIN2A, GATA3 and KDM6A which have been identified in melanoma, breast cancer and recently identified as a candidate driver of pancreatic carcinogenesis, respectively, were only identified within the EUS FNA cytology smear specimens. [24–26] The absolute multigene mutational profile concordance was 83%. This may in part be a reflection upon the tumor microenvironment which is otherwise referred to as desmoplasia or stroma and is comprised of immune cells, macrophages, fibroblasts, myofibroblasts, vascular components and a dense extracellular matrix. [27] Up to 90% of a PDAC tumor mass is composed of the aforementioned desmoplastic stroma which can make the evaluation of tumor cells within a histopathologic specimen challenging. [28] Cytology FNA specimens have the potential to capture more mutations due to the natural concentration of tumor cells in such specimens, as the less cohesive tumor cells may be extracted while the residual dense stromal matrix is excluded. Furthermore, intratumoral heterogeneity represents a variation in tumor behavior between varying sites within the same tumor. We speculate that tumor heterogeneity may have been responsible for the additional mutations detected in 3 cytology specimens. This morphological variation between regions within a tumor has long been familiar to histopathologists but is now gaining increased recognition among clinicians as it may partially account for an impaired treatment response. EUS FNA has the potential to sample cells from a greater number of regions within a tumor than a single FFPE section due to the multiple FNA passes obtained from the tumor in question during an EUS procedure. All of these procedural and sample preparation factors favor EUS FNA as a more sensitive technique for detecting molecular alterations within desmoplastic tumors.

This is a small but fruitful study of archived EUS FNA specimens to illustrate tumor genetic diversity, but if completed on a larger scale could define subgroups with distinct biologic behaviors and even compare and contrast individuals with pancreas adenocarcinoma and separately those with ampullary adenocarcinoma. In an attempt to overcome molecular cytology adequacy challenges, we adhered to strict cytology and FFPE slide screening protocols to qualify samples likely to have a successful NGS. By so doing, it reduced the numbers of patients available to us for evaluation. This therefore prompts the future refinement not only of rapid on site evaluation (ROSE) for cytology adequacy parameters but also corresponding metrics for ROSE molecular adequacy assessments. If such “molecular adequacy” parameters were determined within the procedure room, it could ensure that superior material is obtained for molecular testing than is currently the standard of care for diagnostic purposes only. From a very practical perspective, the turnaround time for molecular diagnostic testing from biopsy procurement to delivery of test results needs to be minimized, if it is to become useful and relevant in a clinical setting. Cancer gene panels are limited to mutation assessment, which although very generous at 160 genes for this particular study, commercial panels most often do not include assessments of chromosomal translocations and copy number variants, which would certainly broaden our understanding of an individual tumor's biologic behavior.

In summary, our study has demonstrated the ability to use a moderate-large targeted NGS cancer gene panel in cytology smear specimens obtained via EUS from PDAC and AA patients as a suitable surrogate for surgically acquired specimens. Such cytology specimens may in fact deliver incremental genetic diversity information. Stratification of patients and targeting of therapy as per NCI initiatives is essential to further expand the developing field of personalized medicine in a truly heterogeneous patient population. The combination of a molecular prognostic and targeted therapy sensitivity grouping may complement the conventional clinicopathologic risk stratification criteria in decision making for clinically based or clinical trial neoadjuvant and adjuvant therapies and guide biomarker driven therapeutic development.

## MATERIALS AND METHODS

### Patient population

Following Mayo Clinic IRB approval, primary malignancy DNA was extracted from 47 chemotherapy naïve patients from archived (2009-2013) molecular cytology single slide smear specimens. The surgical stage pathology grouping was as follows: Ia (7%), Ib (10%), IIa (14%), IIb (62%), III (4%) and IV (3%). Perineural invasion was identified in 13 (44.8%) patients and an R0 status was established in 26 (89.7%) patients. All selected slides had ≥ 20% tumor cells in a background of benign nucleated cells. The use of strict screening criteria allowed the exclusion of 13 patients with insufficient DNA quantity (< 5 ng/μl). ^(9)^ Targeted NGS was performed from the remaining 34 patients, of whom 18 had paired matched surgical pathology specimens with sufficient material for a multigene mutation concordance evaluation.

### DNA extraction process

Cytology smear slides were immersed in xylene for 1-5 days until the coverslip detached. Following rinsing in 95% ethanol, all cellular material from a single slide per patient was scraped with a sterile razor and placed into 1.5 ml tubes. Cytology slide DNA was isolated using the QIAmp DNA Micro kit and FFPE unstained slide DNA was extracted using the QIAamp DSP DNA FFPE Tissue kit (Qiagen Inc, Valencia, CA). DNA samples were quantified using the Qubit® dsDNA BR assay kit (Life Technologies, Carlsbad, CA) as per standard protocol. Extraction yielded 21.0 ng/μl of DNA on average (range 0-88.7) for cytology smear specimens and 66.9 ng/μl of DNA (range 9.3-164) on average for FFPE specimens.

### Deep sequencing of multiplex PCR amplicons

Multiplex PCR was performed by amplifying 10 ng of DNA in each of 4 separate PCR reactions using a commercial Human Comprehensive Cancer GeneRead™ DNAseq Targeted Panel V2 (Qiagen Inc, Valencia, CA) per manufacturer protocol. This panel is a collection of multiplexed PCR primer assays for targeted enrichment of the coding (exonic) regions of the 160 genes (7,951 amplicons) that are most frequently mutated in malignancy with an identifiable oncogenic consequence (Supplementary Table S3).

The PCR products underwent library preparation using the TruSeq Nano DNA Sample Preparation Kit (Illumina, San Diego, CA) as recommended by the manufacturer starting with the end repair reaction. For the Human Comprehensive Cancer Panel, up to 12 samples were pooled equimolar and underwent 2×100bp sequencing on an Illumina HiSeq instrument using the 200 cycle Rapid v2 Reagent Kit (Illumina, San Diego, CA). Internal laboratory studies demonstrated 5-10% analytical sensitivity for mutant alleles at a minimum of 100X coverage.

### Data analysis

Following sequencing completion on the Illumina NGS instrument, the raw sequence reads were extracted and demultiplexed with Illumina CASAVA (Consensus Assessment of Sequence And Variation) program (version 1.8.2) to generate FASTQ files. Sequence FASTQ files were aligned to human genome build hg19 using the CLC BIO Genomics Server (version 6.0) program to produce BAM files. The alignment files were analyzed by the CLC BIO Genomics Server quality and probability variant detection program within a custom bioinformatics pipeline running on the Linux cluster. The Single nucleotide polymorphisms (SNPs) or insertions/deletions (INDELs) with an allele frequency ≥ 5% were manually reviewed and interpreted.

### Statistical analyses

Continuous variables were reported as mean and standard deviation or median and interquartile range and compared by using the Student *t* test or Mann Whitney U test. Categorical variables were reported as frequency (%) and were compared by either a 2-tailed Fisher exact test or Pearson χ^2^ test, where appropriate. Progression free survival (PFS) was defined as the time from EUS FNA to any objective evidence of disease progression or death, whichever occurred first. Cox proportional hazards model was performed to estimate PFS data. All tests were 2-sided, with *P* ≤ .05 as the criterion standard for determining significance. The statistical software package JMP Version 11 (SAS Institute, Cary, NC) and MedCalc version 10 (MedCalc Software, Mariakerke, Belgium) were used for statistical analysis.

## SUPPLEMENTARY TABLES





## Abbreviations

AA: Ampullary adenocarcinoma
COSMIC: Catalogue of Somatic Mutations in Cancer
EUS FNA: Endoscopic Ultrasound Fine Needle Aspiration
FFPE: Formalin fixed paraffin embedded
IPMN: Intraductal Papillary Mucinous Neoplasm
NCI: National Cancer Institute
NGS: Next generation sequencing
PDAC: Pancreatic ductal adenocarcinoma
ROSE: Rapid on site evaluation
WT: Wild type (no somatic mutation detected).
