# Supplementary material for: Targeted next generation sequencing of endoscopic ultrasound acquired cytology from ampullary and pancreatic adenocarcinoma has the potential to aid patient stratification for optimal therapy selection
Source: Oncotarget. 2016 May 18;7(34):54526–36. doi: 10.18632/oncotarget.9440 (PMC5342360; doi:10.18632/oncotarget.9440)
Supplement: Supplementary file 1 [file oncotarget-07-54526-s001.pdf]

Targeted next generation sequencing of endoscopic ultrasound acquired cytology from ampullary and pancreatic adenocarcinoma has the potential to aid patient stratification for optimal therapy selection

SUPPLEMENTARY TABLES

Supplementary Table S1: List of Identified Pathogenic Variants among 21 Genes

See Supplementary File 1

Supplementary Table S2: Spectrum of TP53 Genotype

Mutation	Mutation Type	COSMIC ID
p. Ser215Asn	Missense	COSM44093
p. Val197Met	Missense	COSM43779
p. Phe113Cys	Missense	COSM10717
p. Tyr163Cys	Missense	COSM10808
p. Tyr220Cys	Missense	COSM10758
p. Gln192Ter	Nonsense	COSM10733
c.993+1G>T	Splice Site	COSM6918
p. Cys182Ter	Nonsense	COSM45562
p. Lys132Asn	Missense	COSM43963
p. Arg273Cys x 2	Missense	COSM10659
p. Arg273His	Missense	COSM10660
p. Arg175His	Missense	COSM10648
p. Arg248Gln	Missense	COSM10662
p. Arg337Pro	Missense	COSM378685
p. Gly245Cys	Missense	COSM11081
p. Gly154del	In-frame Deletion	NOVEL
p. Pro190Thr	Missense	COSM44438
p. Pro153AlafsTer28	Duplication/Frameshift	See Note*
p. Pro278Arg	Missense	COSM10887

*Although this specific duplication (c.456dup, Pro153Alafs*28) is not listed in COSMIC, other similar variants with same frame-shift (P153fs*28) have been described (COSM13158, COSM44330, COSM45886, COSM392239).

Supplementary Table S3: Genes evaluated by the Human Comprehensive Cancer GeneRead™ DNAseq Targeted Panel V2

ABL1	BUB1B	DDR2	FGFR2	IDH2	MEN1	PDGFRA	SMARCA4
AKT1	CARD11	DICER1	FGFR3	IKZF1	MET	PHF6	SMARCB1
AKT2	CBL	DNMT3A	FH	IL6ST	MLH1	PIK3CA	SMO
ALK	CBLB	ECT2L	FLCN	IL7R	MSH2	PIK3R1	SPOP
AMER1	CD79A	EGFR	FLT3	JAK1	MSH6	PMS2	SRC
APC	CD79B	EP300	FUBP1	JAK2	MTOR	PPP2R1A	STK11
AR	CDC73	EPCAM	GATA1	JAK3	MUTYH	PRDM1	SUFU
ARID1A	CDH1	ERBB2	GATA2	KDM6A	MYC	PRKAR1A	TERT
ARID2	CDK12	ERBB3	GATA3	KDR	MYD88	PTCH1	TNFAIP3
ASXL1	CDK4	ERBB4	GNA11	KIT	NF1	PTEN	TNFRSF14
ATM	CDKN2A	ERCC5	GNAQ	KLF6	NF2	PTPN11	TP53
ATRX	CHEK2	ESR1	GNAS	KMT2D	NFE2L2	RAC1	TSC1
BAP1	CIC	EZH2	GPC3	KRAS	NFKBIA	RB1	TSC2
BCL6	CREBBP	FAM46C	GRIN2A	MAP2K1	NOTCH1	RET	TSHR
BCOR	CRLF2	FANCA	H3F3A	MAP2K2	NOTCH2	ROS1	U2AF1
BRAF	CSF1R	FANCD2	HIST1H3B	MAP2K4	NPM1	SDHB	VHL
BRCA1	CTNNB1	FANCE	HNF1A	MAP3K1	NRAS	SETD2	WT1
BRCA2	CYLD	FAS	HRAS	MAP4K3	PALB2	SF3B1	XPC
BRIP1	DAXX	FBXO11	HSPH1	MDM2	PAX5	SLC7A8	ZNF2
BTK	DDB2	FBXW7	IDH1	MED12	PBRM1	SMAD4	ZRSR2
