# Supplementary material for: Targeted next generation sequencing of endoscopic ultrasound acquired cytology from ampullary and pancreatic adenocarcinoma has the potential to aid patient stratification for optimal therapy selection
Source: Oncotarget. 2016 May 18;7(34):54526–36. doi: 10.18632/oncotarget.9440 (PMC5342360; doi:10.18632/oncotarget.9440)
Supplement: Supplementary file 2 [file oncotarget-07-54526-s002.docx]

**Supplementary File Table 1**

List of Identified Pathogenic Variants among 21 Genes

| **ID** | **Paired cytology and surgical samples** | **Gene** | **Variant** | **Consequence** | **Cytology variant frequency** | **Matching surgical variant frequency** | **COSMIC ID** |
| --- | --- | --- | --- | --- | --- | --- | --- |
| **9** | No | KRAS | c.35G>A, p.Gly12Asp | missense variant | 29.6 | - | COSM521 |
| **9** | No | TP53 | c.460_462delGGC, p.Gly154del | inframe_deletion | 50.4 | - | - |
| **12** | No | GNAS | c.2530C>T, p.Arg844Cys | missense variant | 35.3 | - | COSM27887 |
| **12** | No | HNF1A | c.341G>A, p.Arg114His | missense variant | 41.6 | - | COSM935970 |
| **12** | No | KRAS | c.35G>A, p.Gly12Asp | missense variant | 36.4 | - | COSM521 |
| **12** | No | TP53 | c.644G>A, p.Ser215Asn | missense variant | 51.7 | - | COSM44093 |
| **15** | No | KDM6A | c.3991C>T, p.Arg1331Ter | stop gained | 57.6 | - | COSM28780 |
| **15** | No | KRAS | c.35G>T, p.Gly12Val | missense variant | 35.2 | - | COSM520 |
| **15** | No | SMAD4 | c.1081C>T, p.Arg361Cys | missense variant | 40.1 | - | COSM14140 |
| **16** | No | KRAS | c.34G>C, p.Gly12Arg | missense variant | 31.2 | - | COSM518 |
| **16** | No | TP53 | c.589G>A, p.Val197Met | missense variant | 6.5 | - | COSM43779 |
| **24** | No | KRAS | c.35G>T, p.Gly12Val | missense variant | 20.2 | - | COSM520 |
| **24** | No | TP53 | c.338T>G, p.Phe113Cys | missense variant | 29.0 | - | COSM10717 |
| **26** | No | KMT2D | c.3392C>T, p.Pro1131Leu | missense variant | 46.7 | - | COSM88089 |
| **26** | No | KRAS | c.35G>A, p.Gly12Asp | missense variant | 14.6 | - | COSM521 |
| **26** | No | TP53 | c.488A>G, p.Tyr163Cys | missense variant | 18.7 | - | COSM10808 |
| **41** | No | KRAS | c.35G>A, p.Gly12Asp | missense variant | 31.9 | - | COSM521 |
| **51** | No | KRAS | c.34G>C, p.Gly12Arg | missense variant | 20.1 | - | COSM518 |
| **51** | No | TP53 | c.574C>T, p.Gln192Ter | stop gained | 38.8 | - | COSM10733 |
| **57** | No | KRAS | c.183A>C, p.Gln61His | missense variant | 59.2 | - | COSM554 |
| **57** | No | TP53 | c.524G>A, p.Arg175His | missense variant | 35.8 | - | COSM10648 |
| **58** | No | KRAS | c.35G>A, p.Gly12Asp | missense variant | 13.1 | - | COSM521 |
| **58** | No | SMAD4 | c.1051dupG, p.Asp351GlyfsTer27 | frameshift variant | 7.2 | - | - |
| **58** | No | SMAD4 | c.1052_1054delATG, p.Asp351del | inframe_deletion | 8.4 | - | COSM1151233 |
| **58** | No | TP53 | c.833C>G, p.Pro278Arg | missense variant | 14.9 | - | COSM10887 |
| **61** | No | FBXW7 | c.1177C>T, p.Arg393Ter | stop gained | 14.7 | - | COSM22973 |
| **61** | No | TP53 | c.568C>A, p.Pro190Thr | missense variant | 37.9 | - | COSM44438 |
| **29** | Yes | KRAS | c.34G>T, p.Gly12Cys | missense variant | 22.7 | 19.4 | COSM516 |
| **29** | Yes | TP53 | c.743G>A, p.Arg248Gln | missense variant | 44.8 | 23.5 | COSM10662 |
| **36** | Yes | TP53 | c.993+1G>T | splice_donor_variant | 55.9 | 32.8 | COSM6918 |
| **37** | Yes | ARID1A | c.5336A>G, p.Glu1779Gly | missense variant | 26.8 | 37.3 | COSM3724472 |
| **37** | Yes | GNAS | c.2530C>T, p.Arg844Cys | missense variant | 28.2 | 23.1 | COSM27887 |
| **37** | Yes | KRAS | c.35G>T, p.Gly12Val | missense variant | 29.1 | 24.8 | COSM520 |
| **37** | Yes | MSH2 | c.1046C>T, p.Pro349Leu | missense variant | 73.5 | 66.2 | - |
| **37** | Yes | NOTCH2 | c.6094C>A, p.His2032Asn | missense variant | 69.4 | 64.5 | COSM1581340 |
| **37** | Yes | TP53 | c.817C>T, p.Arg273Cys | missense variant | 46.5 | 30.6 | COSM10659 |
| **40** | Yes | KRAS | c.35G>A, p.Gly12Asp | missense variant | 39.2 | 22.7 | COSM521 |
| **40** | Yes | TP53 | c.546C>A, p.Cys182Ter | stop gained | 59.4 | 36.8 | COSM45562 |
| **42** | Yes | KRAS | c.35G>T, p.Gly12Val | missense variant | 28.2 | 11.2 | COSM520 |
| **42** | Yes | NOTCH2 | c.6223G>A, p.Val2075Met | missense variant | 54.3 | 51.8 | COSM30497 |
| **43** | Yes | CARD11 | c.1095G>A, p.Met365Ile | missense variant | 9.2 | 1.0 | COSM222114 |
| **43** | Yes | GNAS | c.844G>A, p.Gly282Ser | missense_variant | 5.1 | ND | - |
| **43** | Yes | KRAS | c.35G>A, p.Gly12Asp | missense variant | 5.2 | 5.0 | COSM521 |
| **43** | Yes | RB1 | c.1463C>T, p.Ala488Val | missense variant | 53.9 | 52.3 | COSM254915 |
| **43** | Yes | TP53 | c.524G>A, p.Arg175His | missense variant | 7.0 | 8.2 | COSM10648 |
| **44** | Yes | KRAS | c.35G>T, p.Gly12Val | missense_variant | 36.5 | 11.8 | COSM520 |
| **44** | Yes | PPP2R1A | c.547C>T, p.Arg183Trp | missense variant | 25.4 | 10.6 | COSM51211 |
| **44** | Yes | SMARCA4 | c.2654G>A, p.Arg885His | missense variant | 19.9 | 15.9 | COSM1241759 |
| **44** | Yes | TP53 | c.396G>T, p.Lys132Asn | missense variant | 13.5 | 2.0 | COSM10991 |
| **45** | Yes | KRAS | c.38G>A, p.Gly13Asp | missense variant | 59.5 | 25.8 | COSM532 |
| **45** | Yes | TP53 | c.1010G>C, p.Arg337Pro | missense variant | 73.4 | 14.1 | COSM378685 |
| **47** | Yes | KRAS | c.35G>T, p.Gly12Val | missense variant | 28.8 | 10.5 | COSM520 |
| **47** | Yes | SMAD4 | c.1572G>C, p.Trp524Cys | missense variant | 28.4 | 4.0 | COSM218561 |
| **47** | Yes | TP53 | c.659A>G, p.Tyr220Cys | missense_variant | 31.3 | 10.2 | COSM10758 |
| **48** | Yes | ARID1A | c.1237C>T, p.Gln413Ter | stop gained | 5.5 | ND | - |
| **48** | Yes | ARID1A | c.2989-1G>A | splice_acceptor_variant | 6.0 | ND | - |
| **48** | Yes | GATA3 | c.304G>A, p.Ala102Thr | missense variant | 5.8 | ND | COSM3867910 |
| **48** | Yes | KDM6A | c.1570C>T, p.Gln524Ter | stop gained | 7.2 | ND | COSM4385373 |
| **48** | Yes | KRAS | c.34G>C, p.Gly12Arg | missense variant | 20.6 | 13.6 | COSM518 |
| **48** | Yes | PIK3R1 | c.1057G>A, p.Gly353Arg | missense variant | 31.1 | 13.5 | - |
| **48** | Yes | SMAD4 | c.766C>T, p.Gln256Ter | stop gained | 21.0 | 19.0 | COSM22901 |
| **48** | Yes | TP53 | c.817C>T, p.Arg273Cys | missense variant | 32.2 | 15.6 | COSM10659 |
| **49** | Yes | KRAS | c.34G>C, p.Gly12Arg | missense variant | 8.6 | 10.8 | COSM518 |
| **49** | Yes | SMAD4 | c.1217C>T, p.Ala406Val | missense variant | 10.8 | 7.0 | COSM1389072 |
| **50** | Yes | KRAS | c.182A>T, p.Gln61Leu | missense variant | 38.5 | 14.3 | COSM553 |
| **50** | Yes | SMAD4 | c.1587dupA, p.His530ThrfsTer47 | frameshift variant | 60.4 | 30.7 | - |
| **50** | Yes | TP53 | c.733G>T, p.Gly245Cys | missense variant | 63.4 | 99.6 | COSM11081 |
| **52** | Yes | KRAS | c.35G>T, p.Gly12Val | missense variant | 17.2 | 10.0 | COSM520 |
| **52** | Yes | SLC7A8 | c.1016+1G>A | splice_donor_variant | 47.0 | 48.5 | - |
| **52** | Yes | TP53 | c.672+1G>T | splice_donor_variant | 20.1 | 4.0 | COSM13586 |
| **53** | Yes | KRAS | c.35G>A, p.Gly12Asp | missense variant | 56.3 | 7.4 | COSM521 |
| **53** | Yes | TP53 | c.455dupC, p.Pro153AlafsTer28 | frameshift variant | 76.7 | 50.5 | COSM13158 |
| **54** | Yes | GRIN2A | c.3589G>A, p.Val1197Met | missense variant | 13.9 | ND | COSM1302381 |
| **54** | Yes | KRAS | c.35G>T, p.Gly12Val | missense_variant | 23.3 | 6.8 | COSM520 |
| **55** | Yes | ATM | c.2171delG, p.Gly724ValfsTer11 | frameshift variant | 10.7 | 9.4 | - |
| **55** | Yes | KRAS | c.35G>T, p.Gly12Val | missense variant | 10.4 | 7.4 | COSM520 |
| **55** | Yes | SMAD4 | c.403C>T, p.Arg135Ter | stop gained | 8.9 | 6.0 | COSM14168 |
| **59** | Yes | FANCD2 | c.1077_1078insTGGA, p.Ile360TrpfsTer8 | frameshift variant | 47.7 | 51.8 | - |
| **59** | Yes | KMT2D | c.3737C>T, p.Thr1246Met | missense variant | 22.9 | 21.1 | COSM940080 |
| **59** | Yes | KRAS | c.35G>A, p.Gly12Asp | missense variant | 24.1 | 7.7 | COSM521 |
| **59** | Yes | SMAD4 | c.339dupA, p.Tyr114IlefsTer7 | frameshift variant | 15.0 | 7.5 | - |
| **60** | Yes | KRAS | c.183A>C, p.Gln61His | missense variant | 33.8 | 7.3 | COSM554 |
| **60** | Yes | SMAD4 | c.1217C>T, p.Ala406Val | missense variant | 6.6 | 6.0 | COSM1389072 |

*** ND: not detected**
